# Enhancing Specific Energy and Power in Asymmetric Supercapacitors - A Synergetic Strategy based on the Use of Redox Additive Electrolytes

**DOI:** 10.1038/srep25793

**Published:** 2016-05-17

**Authors:** Arvinder Singh, Amreesh Chandra

**Affiliations:** 1 Department of Physics, Indian Institute of Technology Kharagpur, Kharagpur-721302, West Bengal, India

## Abstract

The strategy of using redox additive electrolyte in combination with multiwall carbon nanotubes/metal oxide composites leads to a substantial improvements in the specific energy and power of asymmetric supercapacitors (ASCs). When the pure electrolyte is optimally modified with a redox additive viz., KI, ~105% increase in the specific energy is obtained with good cyclic stability over 3,000 charge-discharge cycles and ~14.7% capacitance fade. This increase is a direct consequence of the iodine/iodide redox pairs that strongly modifies the faradaic and non-faradaic type reactions occurring on the surface of the electrodes. Contrary to what is shown in few earlier reports, it is established that indiscriminate increase in the concentration of redox additives will leads to performance loss. Suitable explanations are given based on theoretical laws. The specific energy or power values being reported in the fabricated ASCs are comparable or higher than those reported in ASCs based on toxic acetonitrile or expensive ionic liquids. The paper shows that the use of redox additive is economically favorable strategy for obtaining cost effective and environmentally friendly ASCs.

Amongst the electrochemical energy storage devices, supercapacitors (SCs) are at the forefront with their distinctive merits of rapid charging-discharging process, long lifespan, superior durability, high specific power and low maintenance^1^. Nowadays, there are growing research efforts to bring step change in the specific energy of SCs, which is still low in comparison to batteries. The specific energy of the SCs can be improved by enhancing total cell capacitance and by widening the operating cell voltage (E = ½ CV^2^)^2^,^3^. Over the past few decades, most focus has been on the designing of nano-structured electrode materials or use of novel electrolytes for improving the specific energy of the supercapacitor^1^,^2^. It is now clear that supercapacitor geometries and configurations will also have to be investigated to bring step change in performance^3^,^4^. For example, development of asymmetric supercapacitors (ASCs) has seen tremendous growth in recent times^5^,^6^,^7^,^8^,^9^,^10^. Asymmetric supercapacitors bring two different electrode materials together in the same electrolyte in order to extend the operating voltage window of the device. So far, high performance ASCs reported have mostly been fabricated using nano-structured transition metal oxides (TMOs)^11^,^12^,^13^,^14^,^15^. TMOs such as MoO_3_, V_2_O_5_ and WO_3_ with higher work function or electron chemical potential act as hole-injection materials and hold great promise for application as negative electrode materials^16^,^17^,^18^,^19^. In comparison, TMOs like ZrO_2_, MnO_2_ and SnO_2_, etc with low work function or electron chemical potential behave like electron-injection materials and are mostly suitable for positive electrodes^16^,^17^,^18^,^19^,^20^,^21^,^22^. Such TMOs have rich redox chemistry (oxidation/reduction, intercalation/de-intercalation, chemisorption, etc.) but overcoming their limited specific power remains a challenge^23^,^24^,^25^. As a result, composite of TMOs with multiwall carbon nanotubes (MWCNTs) is becoming popular^26^,^27^,^28^,^29^. The presence of conducting MWCNTs not only provides channels for electron conduction but also introduce mesoporosity to the composites. These two features play an important role for achieving high capacitance in SCs.

The operating voltage window of an asymmetric cell is a convoluted effect of overpotential provided by the electrolytes and the difference of work functions of negative (Φ_n_) and positive (Φ_p_) electrodes i.e., Φ_n_ − Φ_p_^16^,^17^,^30^. Therefore, ASCs fabricated using TMOs with a large difference in their respective work functions and neutral aqueous electrolytes (having highly solvated ions) may be operated up to voltages as high as 2.2 V. The methodology of carefully unbalancing the device has also been recently proposed to increase the operating voltage window^31^,^32^. In this paper, we show a novel strategy i.e., use of optimized concentration of redox additive electrolyte to bring significant enhancement in the specific energy whilst maintaining power of asymmetric supercapacitors. Very few studies have been undertaken to explore the use of redox additives in the 3-electrode or symmetric cells^33^,^34^,^35^,^36^. In these reports, galvanostatic charge-discharge curves are highly distorted exhibiting either a wide plateau region and/or non-linearity within a given discharge voltage range. The reported specific power and energy values are also overestimated since for such charge-discharge profiles, simply *dV/dt* (slop) cannot be considered. We report that with the use of much lower concentrations of redox additives, a transition from wide plateau region to linear and symmetrical charge-discharge profiles could be achieved. The ASCs discussed in this work were fabricated using neutral aqueous electrolyte, high surface area mesoporous MWCNTs/ZrO_2_ (MWZ) and MWCNTs/WO_3_ (MWW) composites, as positive and negative electrodes, respectively. It is shown that the charge-balanced ASCs can be operated up to 2.2 V leading to specific energy and power as high as ~65 Wh kg^−1^ and ~950 W kg^−1^, respectively. The specific energy value is significantly enhanced on addition of the optimized quantity of redox additive viz., potassium iodide (KI). More specifically, increase of ~105% in the specific energy value was observed with good cyclic stability even after 3,000 charge-discharge operations. With such high specific energy and power values, the proposed ASCs have the capacity for large scale integration in applications such as portable electronics devices, back-up power supplies, hybrid electric vehicles and energy harvesting devices.

## Results

### Physical characterizations of materials

**Figure 1:**
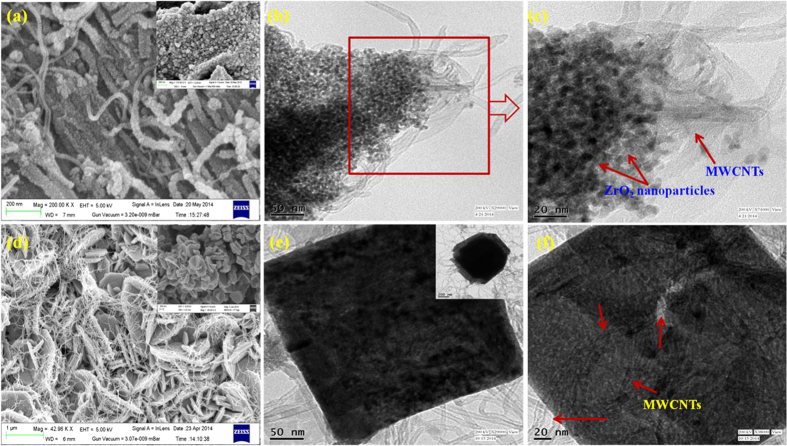
FESEM and TEM micrographs observed for (**a–c**) MWZ. (**d–f** ) MWW composite materials. Inset to (**a,d**) represent FESEM micrographs for ZrO_2_ and WO_3_.

A detailed chemical route used for the synthesis of MWZ and MWW composites is illustrated in Supplementary Fig. S1. Phase purity of the cubic ZrO_2_ (JCPDS file #27–997) and monoclinic WO_3_ (JCPDS file #32–1395) in the MWZ and MWW composites, respectively was confirmed by the analysis of XRD data (see Supplementary Fig. S2). Nanostructure morphologies and homogeneity in particle size distribution is highly desirable for supercapacitor electrode materials. From the FESEM and TEM micrographs (Fig. 1a–f), it is clear that MWW comprises of WO_3_ nanostructures (constructed from >300 nm thick plates) and partially disentangled MWCNTs lying underneath these nano-plates. On the other hand, MWZ possessed nano-sized ZrO_2_ particles attached to the surface of MWCNTs (MW). The uniform distribution of ZrO_2_ and WO_3_ nanostructures in the composite systems was easily discernible by the analysis of focus ion beam (FIB) elemental maps (see Supplementary Fig. S3). Thermogravimetric analysis (TGA) data returned the percentages of WO_3_ and ZrO_2_ in the MWW and MWZ composites as ~80 and 75 wt%, respectively (see Supplementary Fig. S4).

**Figure 2:**
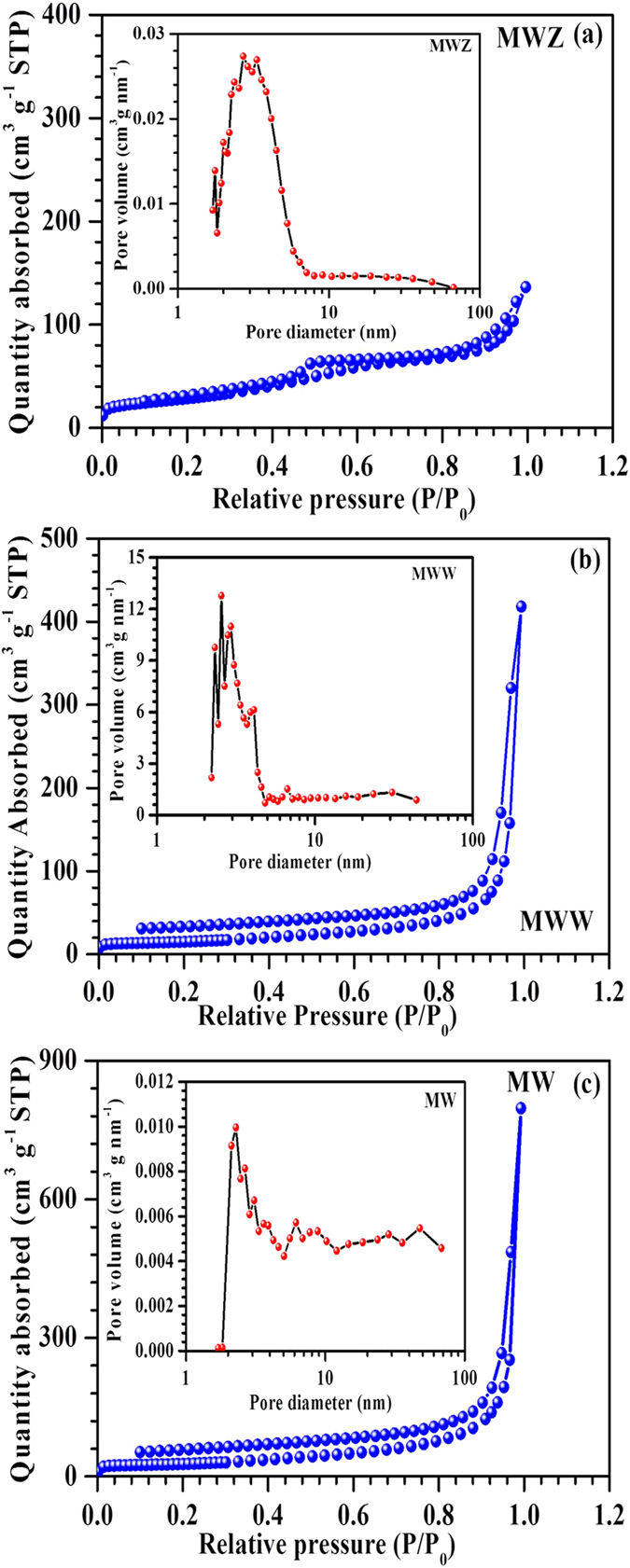
N_2_ absorption-desorption isotherms and pore size distribution observed for: (**a**) MWZ, (**b**) MWW composite materials and (**c**) MWCNTs (MW).

In composites, the electrochemical response is directly associated with the surface area and porosity that becomes available for chemical reactions and/or charge intercalation/de-intercalation. These two parameters are routinely determined using the N_2_ absorption-desorption isotherms which, for the composites and MWCNTs, are shown in Fig. 2a–c. The occurrence of Type IV isotherms in all the three samples indicated the presence of meso-porosity. The Brunauer-Emmett-Teller (BET) surface areas, total pore volume and BJH desorption average pore diameter for MWZ, MWW and MWCNTs were found to be: 103.8, 51.3 and 92 m^2^ g^−1^, 0.2108, 0.6467 and 1.2331 cm^3^ g^−1^ and 5.47, 24.68 & 19.41 nm, respectively. The peak below 10 nm observed in the pore size distribution curves for all the materials further point towards the majority of mesopores.

### Three electrode cyclic voltammetry analysis and charge-balancing

The merits of the obtained MWZ and MWW composites over their constituents (MWCNTs, WO_3_ or ZrO_2_) for use in ASCs became evident after the 3-electrode cyclic voltammogram (CVs) were collected in 1 M Li_2_SO_4_ aqueous electrolyte with a three-electrode system consisting a Pt counter electrode and a saturated KCl Ag/AgCl reference electrode (see Supplementary information Fig. S5). Supplementary Fig. S5a compares CVs for the MWCNTs, ZrO_2_ and MWZ at a scan rate of 50 mV s^−1^ in a positive potential (0–1.1 V) range. The higher storage capacity of the composite system immediately becomes evident; since the area under CVs at a given scan rate is directly proportional to charge storage capacity. The improved capacity is a consequence of the synergistic interaction between highly conducting MWCNTs and chemically active ZrO_2_ nanostructures. The charge storage mechanisms in the system was a convolution of additive effects originating from the intercalation/de-intercalation (ZrO_2_ + *δ*M^+^ + *δ*e^−^ ↔ M_*δ*_ZrO_2_) and surface absorption/desorption ((ZrO_2_)_surface_ + M^+^ + e^−^ ↔ (ZrOOM)_surface_) of the electrolyte cations (M^+^)^37^. As MWZ composite acts like a positive electrode, during charging Li^+^ is driven out while in the discharging cycle, intercalation of Li^+^ into the ZrO_2_ mesoporous structures takes place.

The cyclic voltammetry (CVs) curves were also recorded to compare the charge storage capacities of the MWCNTs, WO_3_ and MWW composite in a wide negative potential range (−1.1 to 0 V). The data is given in Supplementary Fig. S5b. Highest capacitive behaviour was observed for the MWW composite using the CVs data analysis paved way for its use as a negative electrode. This meant that, in MWW, intercalation of Li^+^ would occur during charging while discharging will force de-intercalation of Li^+^ ions^38^. The CVs for MWZ and MWW at different scan rates were also recorded in their respective potential ranges and are shown in Supplementary Fig. S5c,d. The MWZ (MWW) composite showed a maximum specific capacitance of ~600 F g^−1^ (~720 F g^−1^) at a scan rate of 10 mV s^−1^, which expectedly decreased to ~435 F g^−1^ (~408 F g^−1^) at 200 mV s^−1^.

To have a charge-balanced device, the desired mass ratio between positive and negative electrodes was estimated using the mass-balance relation given as:


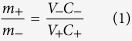


where *m*_+_ (*m*_*−*_) is the mass of active material, *C*_+_ (*C*_*−*_) is the capacitance at same scan rate and *V*_+_ (*V*_*−*_) is the potential range for positive (negative) electrode material. For the present case, the value of ratio was ~1.2.

### Fabrication of asymmetric devices and their electrochemical performance

**Figure 3:**
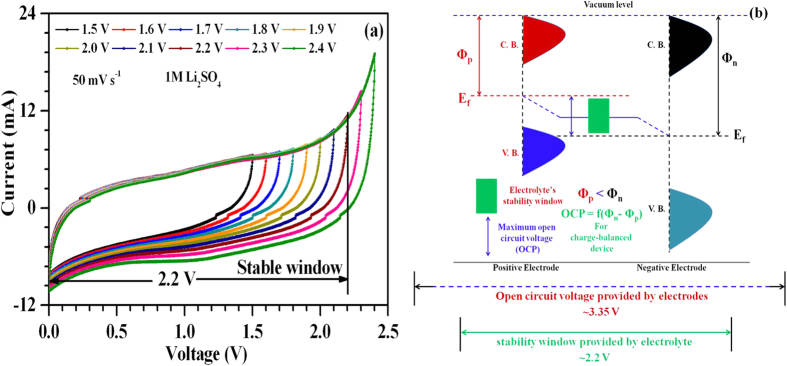
(**a**) Two electrode CV curves observed in different voltage ranges at 50 mV s^−1^ for ASCs assembled in 1 M Li_2_SO_4_ electrolyte and (**b**) Energy band diagram to explain maximum achieved operating voltage window.

To test the electrochemical performance of the synthesized composite materials in asymmetric device, Hohsen 2032 type coin cells (outer diameter 20 mm) were assembled using positive and negative electrodes (with desired mass ratio estimated from mass-balance equation at 50 mV s^−1^ i.e., *m*_+_/*m*_−_ = 1.2; with *m*_+_ = 1.2 mg and *m*_−_ = 1.0 mg) and Whatman glass microfiber filters (GF/C grade) pre-soaked in 1 M Li_2_SO_4_ aq. electrolyte systems. CVs of ASCs in different voltage ranges were collected and are shown in Fig. 3a. The ASCs showed stable operation up to 2.2 V. The operating voltage window of a device can be given as:


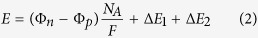


where *Φ*_*n*_ and *Φ*_*p*_ are the work functions for the positive and negative electrodes, while *N*_*A*_ and F represent the Avogadro’s number and Faraday constant. Δ*E*_*1*_ and Δ*E*_*2*_ are the electrode potentials for the positive and negative electrodes, respectively^16^,^17^,^30^,^39^,^40^,^41^. Therefore, for the charge-balanced ASCs, the maximum operating voltage window is strongly governed by (a) difference in the work functions (i.e., *Φ*_*n*_ − *Φ*_*p*_) of the electrodes and (b) decomposition energy of the solvent. In d^0^ type oxides viz., ZrO_2_ and WO_3_, used in the present studies, the work function difference of ~3.35 eV has been reported earlier^17^,^42^. The obtained voltage window is lower than the value (~3.35 V) which can be theoretically estimated from equation (2). This decrease can be due to chemisorption of H^+^ (OH^−^) species on the negative (positive) electrode that modifies the work function difference^17^. Also, the neutral aqueous (i.e., 1 M Li_2_SO_4_) electrolyte can provide maximum voltage up to 2.2 V without H_2_/O_2_ evolution due to highly hydrated lithium cation and sulfate anions i.e., ions with high hydration energy^43^. Therefore, in the present case, maximum OCP was limited to 2.2 V by the decomposition energy of the water. This is schematically explained by an energy band diagram in Fig. 3b.

ASCs were further characterized using cyclic voltammetry and galvanostatic charge-discharge studies (see Supplementary information Fig. S6). The observed CV curves at different scan rates between 0–2.2 V for ASCs assembled in 1 M Li_2_SO_4_ electrolyte are given in Fig. S6a. At each scan rate, ASCs shows nearly horizontal and roughly rectangular-shaped CVs suggesting good capacitive behaviour of the devices with significant contribution from the series resistance. The possible redox reactions occurring at the positive and negative electrodes of ASCs are as follows:


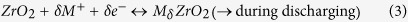



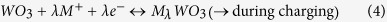


the galvanostatic charge discharge measurements performed at different specific currents for the ASCs are given in Fig. S6b. The ASCs shows linear and symmetrical charge-discharge profiles indicating high coulombic efficiency (~94%) of these devices. The obtained maximum specific capacitance for ASCs was ~96 F g^−1^ at 1 A g^−1^; reduced to ~58 F g^−1^ at 10 A g^−1^ with ~60% capacitance retention indicating good rate capability of these devices. The observed specific capacitance is much lower than those observed for individual composites from CV curves in 3-electrode system. This is because (a) 3-electrode measurements (w. r. t. reference electrode; negligible effect of ‘IR’ drop) provide four times higher capacitance than what is measured in a two electrode system, and (b) due to the different time-scales of these two (cyclic voltammetry and charge-discharge) techniques^44^,^45^,^46^. The maximum specific energy was found to be ~65 Wh kg^−1^ at a specific power of ~950 W kg^−1^. The high specific energy obtained for the fabricated ASCs in 1 M Li_2_SO_4_ electrolyte system can be explained by (a) the presence of high surface area mesoporous composite materials. The larger mesopores provide channels for ion diffusion with short diffusion length while small mesopores mainly contribute for charge storage. Further, high surface area enables a large interfacial contact region. This results in total cell capacitance for correctly charge-balanced ASCs as high as ~96 F g^−1^ at 1 A g^−1^ and (b) a large operating voltage window of 2.2 V. These features endow ASCs to show specific energy as high as ~65 Wh kg^−1^. The obtained specific energy is comparable or higher to most of the values reported previously for ASCs based on aqueous electrolytes^7^,^10^,^12^,^15^,^17^,^47^,^48^,^49^,^50^,^51^. Therefore, another strategy has to be adopted to bring a quantum jump in the specific energy.

### Asymmetric supercapacitors in redox additive electrolyte

**Figure 4:**
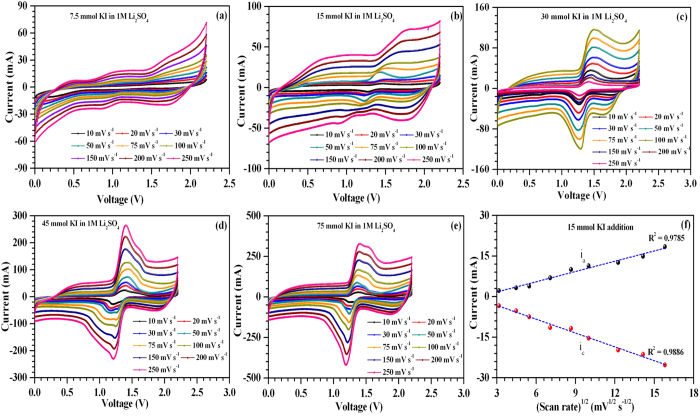
Two electrode CV curves observed at different scan rates for ASCs assembled after addition of (**a**) 7.5 mmol (**b**) 15 mmol (**c**) 30 mmol (**d**) 45 mmol and (**e**) 75 mmol KI in aq. 1 M Li_2_SO_4_ electrolyte, resp-ectively (**f** ) Variation of anodic and cathodic peak currents with v^1/2^ (v is scan rate) for ASCs assembled in 15 mmol KI added electrolyte system.

**Figure 5:**
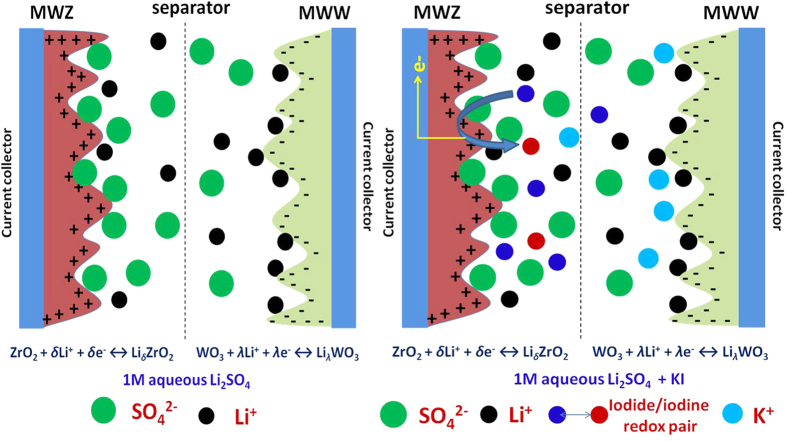
Schematic showing the various charge-storage processes occurring at the electrode/electrolyte interface in an ASC.

**Figure 6:**
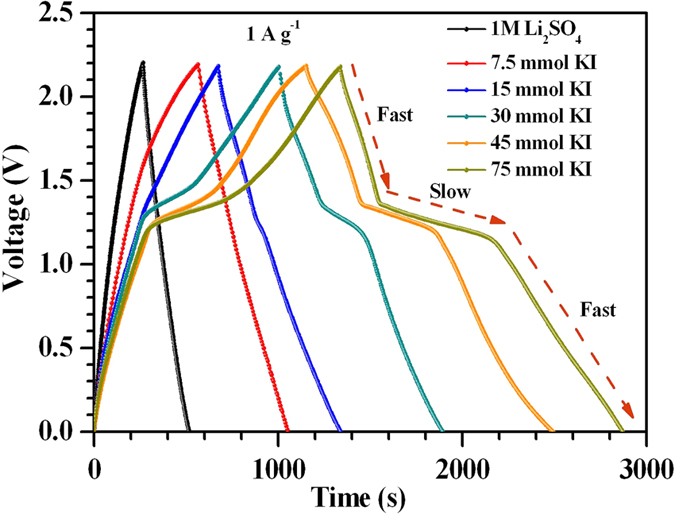
Galvanostatic charge-discharge curves at 1 A g^−1^ for ASCs fabricated in only 1 M Li_2_SO_4_ and after addition of different KI concentrations.

For the aim of reaching much higher specific energy or power without using toxic or expensive electrolytes such as acetonitrile or ionic liquids, the use of redox additive is proposed. The redox additives are expected to contribute synergistically in ionic conductivity and the total capacitance value of the cell. The CV curves for the ASCs fabricated using 1 M Li_2_SO_4_ and KI redox additive (varying concentrations) are shown in Fig. 4. The presence of highly distorted CV profiles along with the redox peaks indicated the presence of both double layer and pseudo/Faradaic capacitance. Such Faradaic capacitance occurs due to various redox reactions of iodine/iodide redox pairs. The possible oxidation/reduction reactions that can occur at the electrode/electrolyte interface due to these iodide/iodine redox pairs can be written as:


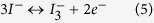



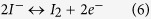



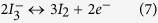



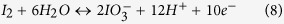


the nearly linear increase in the anodic (*i*_*a*_) and cathodic peak (*i*_*p*_) currents as a function of square root of scan rates (see Fig. 4f) indicates the quasi-reversibility of these redox reactions with diffusion limited processes. As the electrode materials have a mesoporous structures, the solvated iodine species (such as polyiodides) of size ~1.8 nm can also intercalate and accumulate inside the surface^33^,^34^. The overall double-layer/redox processes occurring at each electrode/electrolyte interface in the ASCs is schematically shown in Fig. 5. The galvanostatic charge-discharge profiles obtained at 1 A g^−1^ for the ASCs fabricated with and without KI additions are shown in Fig. 6. At higher concentrations of KI, the charge-discharge profiles exhibited a wide plateau region thus giving three different power regions (i.e., different *dV/dt*). This plateau region is appeared due to large number of redox reactions of iodine/iodide redox pairs occurring at the electrode/electrolyte interface. This suggested the strong contribution from the pseudo/Faradaic capacitance at higher KI concentrations. Therefore, power of the ASCs is deteriorated at higher KI concentrations.

**Figure 7:**
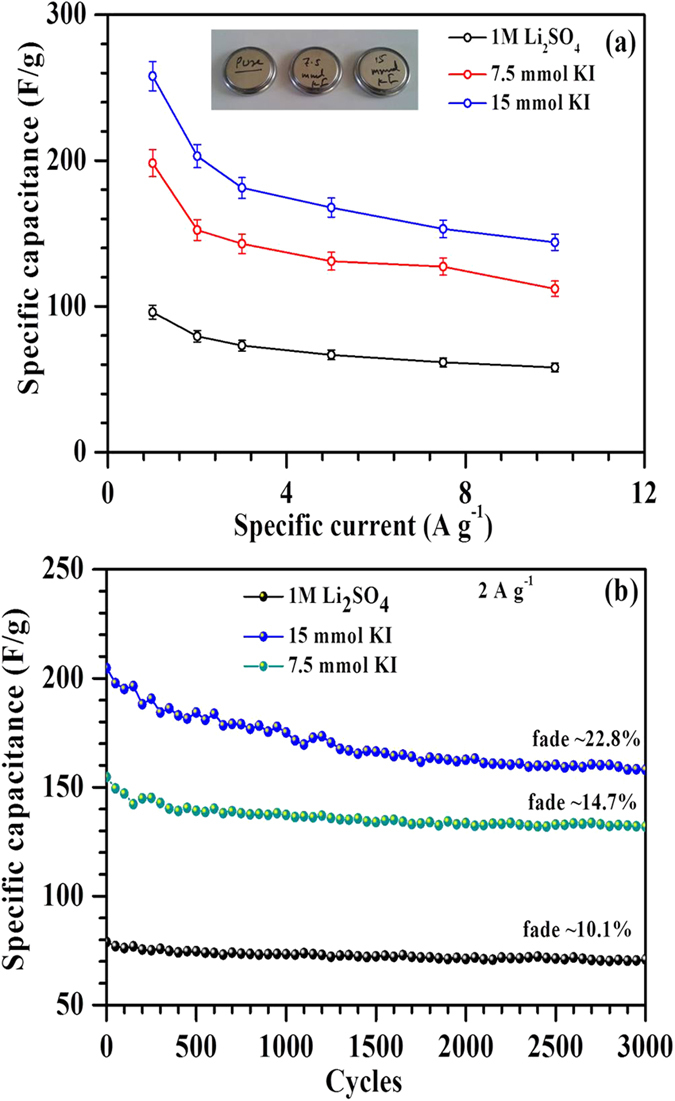
(**a**) Rate capability and (**b**) Cycling stability of ASCs in only 1 M Li_2_SO_4_ and with addition of KI (7.5 and 15 mmol).

**Figure 8:**
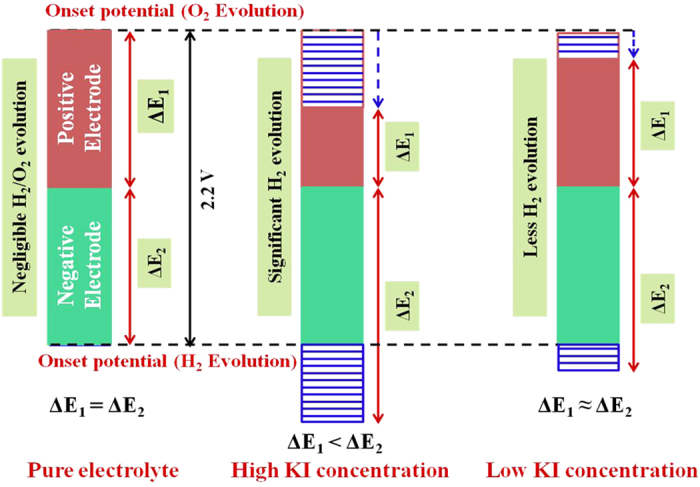
A schematic diagram to explain the reduction of cyclic stability at higher KI concentrations.

For practical applications, symmetrical and linear charge-discharge profiles are desirable. It was found that ~7.5 mmol KI gave the favorable charge-discharge profiles amongst all KI concentrations (see Supplementary information Fig. S7). At this concentration, substantial increase (~105%) in the specific energy could be obtained. Table 1 summarizes the morphology, BET surface area, individual capacitance of the composites in 3-electrode system and capacitance of the ASCs assembled with and without addition of KI. Moreover, the ASCs fabricated with only Li_2_SO_4_ or with addition of 7.5 mmol KI showed good rate capability with more than 50% capacitance retention at higher specific currents (see Fig. 7a). As desired, in devices, the ASCs with 7.5 mmol redox additive (KI) concentration also had good cycling stability with only ~14.7% capacitance fade after 3,000 charge-discharge operations (Fig. 7b). The reduction in cyclic stability with increasing KI concentration can be explained using Fig. 8. As the concentration of KI increases, stable potential window of the positive electrode tends to shrink due to large number of redox reactions of I^−^ ions at the positive electrode/electrolyte interface^52^. This will lead to an ASC with a charge imbalanced state due to unequal voltage splitting at positive and negative electrodes. Therefore, H_2_ generation will start at the negative electrode and results in higher capacitance fade (~22.8% for 15 mmol in Fig. 7b).

**Figure 9:**
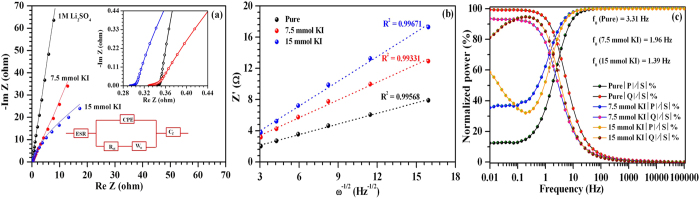
(**a**) Nyquist plot, (**b**) Randles plot and (**c**) Complex power analysis for ASCs fabricated in 1 M Li_2_SO_4_ aqueous electrolyte and after the addition of KI (7.5 and 15 mmol).

To further understand these electrochemical characteristics and charge-storage kinetics, ASCs were examined using the electrochemical impedance spectroscopy (EIS) study. Figure 9a shows the typical Nyquist plot observed for ASCs assembled using only Li_2_SO_4_ and KI added (7.5 and 15 mmol) electrolyte systems. The equivalent series resistance (ESR) for the ASCs assembled in only Li_2_SO_4_ and with addition of 7.5 and 15 mmol KI concentration were found to be 344, 330 and 290 mΩ, respectively. These originate from the resistance produced by the bulk electrolyte, current collectors, contacts and the electrode materials. Intriguingly, the ESR reduces slightly with increasing KI concentration. This indicates improved ionic conductivity of the electrolyte system. For all the ASCs, a semicircle was observed in the high frequency region, representing a charge transfer region at electrode/electrolyte interfaces. The diameter of the semicircle tends to increase at higher KI concentration. Thus, suggesting an enhanced charge-transfer resistance. The deviation from ideal capacitor behaviour at lower frequencies can be attributed to the distributed macroscopic path lengths of the electrolyte ions inside the porous electrodes^53^. Further, region at about 45°, in the moderate frequency range, arises due to frequency dependent diffusion of the electrolyte ions inside the porous electrodes. These Nyquist plots can also be represented by an equivalent circuit as shown in an inset to Fig. 9a, where CPE is the constant phase element, *R*_*ct*_ and *W*_*o*_ are frequency dependent components known as charge transfer resistance and Warburg element and *C*_*f*_ is the pseudocapacitive element. Supplementary Table S1 shows the various parameters obtained from the fitting of the Nyquist plots. The Warburg impedance can be expressed as follows:


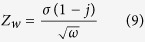


where *ω* = 1/2π*f, j* is imaginary number and *σ* is Warburg coefficient^54^. The Warburg coefficient *σ* is a function of temperature, diffusion coefficient of ionic species and bulk electrolyte concentration^54^. This can be estimated from the slope of the Randles plot (i.e., *ω*^−1/2^ *vs. Z’*) as shown in Fig. 9b. It was observed that the Warburg impedance *Z*_*w*_ increased at higher KI concentration. This is a direct consequence of the reduced diffusion coefficient (as *Z*_*w*_ is inversely proportional to square root of diffusion coefficient) due to the large stoke/solvation radius of the solvated iodide ions resulting in lower mobility^54^. Therefore, ASCs with higher KI concentrations reflect relatively low specific power at lower frequency.

The relaxation time τ_0_ was also estimated by complex power analysis. Figure 9c shows variation of normalized active (*|P|*) and reactive (*|Q|*) powers as a function of frequency for the ASCs. It can be seen that, below a critical relaxation frequency f_0_ (relaxation time *τ*_*0*_ = 1/*f*_0_), ASCs assembled in only Li_2_SO_4_, exhibited constant power characteristics whereas ASCs having redox additive i.e., 15 mmol KI, showed reduced power at lower frequency (i.e. 10 mHz). The low concentration of KI allows the electrode materials to uptake and release I^-^ ions comparatively at a faster rate. Consequently, as mentioned before, low (7.5 mmol) KI concentration is more suitable for maintaining the high power characteristics of these ASCs.

**Figure 10:**
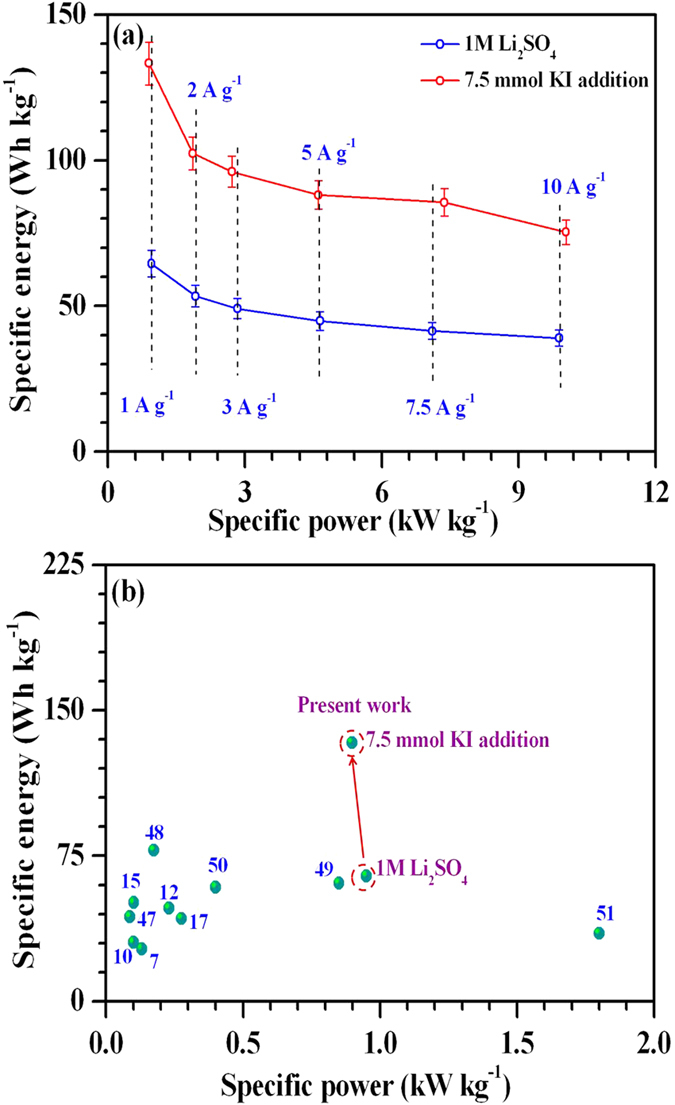
(**a**) Ragone plot and (**b**) Performance comparison of proposed ASCs with previously reported laboratory scale ASCs in aqueous electrolyte.

Figure 10a represents Ragone plot for ASCs fabricated using only Li_2_SO_4_ and with addition of 7.5 mmol KI. It is clear that specific energy of the device is significantly improved at 7.5 mmol KI concentration with negligible loss in specific powers (indicated by the vertical dotted lines) at each specific current. ASCs with 7.5 mmol KI showed highest specific energy of ~133 Wh kg^−1^ at a specific power of ~898 W kg^−1^. This reduced to ~75 Wh kg^−1^ at 10 A g^−1^ while specific power increased to ~10,036 W kg^−1^. Although, values quoted for specific capacitance, energy and power were estimated for similar small laboratory scale electrodes with consideration of mass of the active materials only, these are superior to those reported previously for small laboratory scale aqueous ASCs (Fig. 10b)^7^,^10^,^12^,^15^,^17^,^47^,^48^,^49^,^50^,^51^. A more detailed comparison including device structure, operating voltage, cycling and Ragone features between our and previously reported laboratory scale ASCs is also given (see Supplementary information Table S2). The performance comparison is made with only those ASCs where the mass loading of the active materials was nearly the same (i.e. ≤1.5 mg cm^−2^). We have also performed galvanostatic charge-discharge measurements for the ASCs fabricated with higher mass loading of the active materials (m = ~6.6 mg; m_+_ = ~3.6 mg, m_−_ = ~3 mg) and are shown in Supplementary Fig. S8. The maximum specific capacitance for the ASCs in pure electrolyte could reach ~30 F g^−1^ (corresponding specific energy ~20 Wh kg^−1^), which increased to ~55 F g^−1^ (corresponding specific energy ~33 Wh kg^−1^) when the electrolyte is slightly modified with 7.5 mmol KI. These results clearly show the potential of redox additives in ASCs even at higher mass loadings.

## Discussion

The use of high surface area mesoporous MWCNTs/ZrO_2_ (WO_3_) composites resulted in a high total cell capacitance of ~96 F g^−1^. The operating voltage window could reach as high as 2.2 V due to high overpotential provided by the neutral aqueous electrolytes. These features enable ASCs to show high specific energy of ~65 Wh kg^−1^ at 1 A g^−1^ specific current. The improved capacitance in ASCs with addition of KI is due to the presence of iodine/iodide redox pairs, which can further form variety of polyiodides such as I_3_^−^, I_5_^−^ and IO_3_^−^ through dissolved I_2_. This is the unique aspect of iodide based additives that the product i.e. polyiodides (I_n_^−^) are also negatively charged. Therefore, these polyiodides can also function, similar to counter-ions, for charge balancing in the EDL via electro-sorption at positively charged electrode. But, the iodine/iodide redox pairs originate enormous capacitance only at the positive electrode of ASCs. Moreover, at high KI concentrations, stable potential window of the positive electrode tends to shrink and results in H_2_/O_2_ evolution at a lower potential than 2.2 V. Consequently, ASCs with high KI concentration exhibited undesired power characteristics and relatively more capacitance fade (~22.8% for 15 mmol KI in Fig. 7b). At 7.5 mmol KI concentration, all these effects are found to be minimal thus allowing ASCs to show linear and symmetrical charge discharge profiles and relatively good cyclic stability. It is clearly evident that great attention needs to be paid while choosing concentration of KI in order to avoid detrimental effects on the specific power and cycling stability of ASCs.

## Conclusions

It is clearly demonstrated that fabrication of ASCs with an optimized concentration of redox additive to a neutral aqueous electrolyte will lead to an increase of specific energy whilst maintaining power values. Along with ~105% increase in specific energy at 7.5 mmol KI concentration, iodine based redox reactions can also ensure good cyclic stability and high specific power. The fabricated ASCs also used the advantages of (a) high surface-area mesoporous TMOs/MWCNTs composites and (b) a wide operating voltage window. This work provides useful information on how the redox additives can potentially be used in ASCs for simultaneously achieving high specific energy and power.

## Methods

### Chemical used

Multiwall carbon nanotubes (MWCNTs) (ID 3–5 nm; OD 20–25 nm; length 20 μm and purity 95.0%, Nanocyl, Belgium), were purchased from Nanocyl (Belgium). Zirconium oxychloride octahydrate (ZrOCl_2_.8H_2_O), and sodium tungstate dehydrate (Na_2_WO_4_.2H_2_O), Loba Chemie Pvt. Ltd., India were used as starting raw materials with desired stoichiometry. Whatman glass microfiber filters (GF/C^TM^; diameter 47 mm) were purchased from GE Healthcare UK Limited, UK.

### Material synthesis and their characterizations

For the synthesis of electrode materials, first, MWCNTs were refluxed in concentrated HNO_3_ (69%) at 120 °C for 12 h to induce surface functionalization. For the MWZ composite, 200 mg functionalized MWCNTs were dispersed in 300 ml ZrOCl_2_.8H_2_O aqueous solution (0.05 mol/L in DI water) using ultrasonication. Excess ammonia solution (25%) was next added to the dispersion and entire mixture was stirrered vigorously at 80 °C for 5 h in an oil bath. The product was filtered, washed several times with DI water and vacuum dried at 80 °C for 12 h. MWZ composite was prepared by calcining the collected product at 550 °C in N_2_ environment. To synthesize MWW composite, 200 mg functionalized MWCNTs and 2.5 g of Na_2_WO_4_.2H_2_O were mixed in 300 ml DI water. Then, 3 M HCl was added dropewise and mixture was stirrered at 95 °C for 4 h in an oil bath. Subsequently, product was filtered, washed several times with DI water and vacuum dried at 80 °C for overnight. MWW was obtained by calcining the obtained product at 350 °C for 5 h in N_2_ environment with a temperature ramp rate of 50 °C/min. The ZrO_2_ and WO_3_ samples were also synthesized separately by following the same method in the absence of MWCNTs.

Powder X-ray diffraction (XRD) spectra were collected in the 2*θ* range 15–90° for all the materials using PAN Analytical diffractometer with Cu-Kα (*λ* = 1.5406 Å) as the incident wavelength. For morphological studies, the samples were investigated using field emission scanning electron (SEM CARL ZEISS SUPRA 40) and transmission electron (TEMFEI-TECHNAI G220S-Twin operated at 200 kV) micrographs. Thermogravimetric analysis (TGA) was performed in O_2_ environment at 10 °C min^−1^ by NETZSCH STA 409 PC/PG thermal analyzer. The Brunauer- Emmett-Teller (BET) surface area and porosity was measured by analyzing adsorption-desorption isotherms obtained from Micromeritics Gemini V Model 2365 and Gemini VII Model 2390t.

### Device fabrication and electrochemical characterizations

Initially, 95% of the active materials (i.e., MWZ, ZrO_2_, WO_3_, MWCNTs or MWW) and 5% polyvinylidene fluoride (PVDF) were stirrered in 50 ml acetone and heated at 100 °C to get homogeneous stable slurry. The slurry was then deposited on a commercially available graphite sheet (100 μm thick) using film coater. Finally, electrodes were cut to assemble coin cells and dried at 100 °C.

All the electrochemical measurements i.e., cyclic voltammetry (CV), galvanostatic charge-discharge and electrochemical impedance spectroscopy (EIS) were performed using Metrohm Autolab (Galvanostat/Potentiostat). A three-electrode system was used to record cyclic voltammogram (CV) for all the active materials in 1 M Li_2_SO_4_ aq. electrolyte, where Pt was used as counter electrode and Ag/AgCl (sat. KCl) was used as a reference electrode. For the working electrode, active materials were coated on graphite sheets (which works as a current collector). The specific capacitance from the CV curves was calculated using the relation:


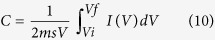


where *m* is the mass of active material excluding mass of the binder (here *m* ~ 1 mg for all the materials in 3-electrode measurements), *s* is the scan rate in mV/s, *V*_*i*_ and *V*_*f*_ represent the lower and upper voltage value for voltage window range V, and I(V) is the current response. To test the electrochemical performance of the synthesized composite materials in asymmetric device, Hohsen 2032 type coin cells (outer diameter 20 mm) were assembled using positive and negative electrodes (with desired mass ratio estimated from mass-balance equation at 50 mV/sec i.e., *m*_+_/*m*_−_ = 1.2; with *m*_+_ = 1.2 mg and *m*_*−*_ = 1.0 mg) and Whatman glass microfiber filters (GF/C grade) pre-soaked either in pure or KI added 1 M Li_2_SO_4_ aq. electrolyte systems. These cells were characterized by CV, galvanostatic charge-discharge and EIS techniques. The specific capacitance from the charge-discharge profiles was estimated using following equation:


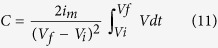


where *i*_*m*_ is the constant specific current in A g^−1^ (here mass of both the active materials is taken; *m* = 2.2 mg to calculate applied *i*_*m*_), integration of ‘*Vdt*’ is the area under the discharge curves while *V*_*f*_ and *V*_*i*_ representing final and initial values of the voltage range. The average specific energy and power for the as-fabricated coin cells were estimated by employing the formula:


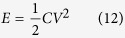



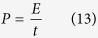


where *E* is the specific energy, *V* is the discharging voltage range excluding *IR* drop, *P* is the specific power and t is the discharging time.

The variation of real (*|P* (*ω*)*|*/*|S* (*ω*)*|*) and imaginary part (*|Q* (*ω*)*|*/*|S* (*ω*)*|*) of the normalized complex power *S* (*ω*) as a function of frequency were estimated using the relations:


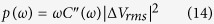



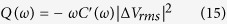



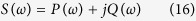


where *|*Δ*V*_rms_*|*^2^ = Δ*V*_max_/√2 (*V*_max_ is the maximum amplitude of the applied ac voltage; here 5 mV) and *j* is imaginary number while *ω* refers to the angular frequency and equal to 2π*f*. The *C*′ and *C*′′ are the real and part of the complex capacitance calculated from the relations as given below:


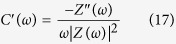



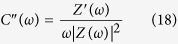


where *Z*′ (*ω*) and *Z*′′ (*ω*) are the real and imaginary parts of the complex impedance *Z* (*ω*).

## Additional Information

**How to cite this article**: Singh, A. and Chandra, A. Enhancing Specific Energy and Power in Asymmetric Supercapacitors - A Synergetic Strategy based on the Use of Redox Additive Electrolytes. *Sci. Rep.* **6**, 25793; doi: 10.1038/srep25793 (2016).

## Supplementary Material

Supplementary Information

## Figures and Tables

**Table 1:** Summary of sample’s morphology, electrolytes, BET surface area and capacitance values.

**Sample/Device**	**Morphology**	**BET surface area (m**^**2**^**/g)**	**Electrolyte**	**Scan rate/specific current**	**Capacitance (F/g)**
**MWCNTs/ZrO**_**2**_ **(MWZ); 3-electrode**	ZrO_2_ nanoparticles/ MWCNTs	103.8	1 M Li_2_SO_4_	10 mV/s	~600
**MWCNTs/WO**_**3**_ **(MWW); 3-electrode**	WO_3_ nanostructures/ MWCNTs	51.3	1 M Li_2_SO_4_	10 mV/s	~720
**ASCs 2-electrode**	–	–	1 M Li_2_SO_4_	1 A/g	~96
**ASCs 2-electrode**	–	–	1 M Li_2_SO_4_ + 7.5 mmol KI	1 A/g	~198
