# Supplementary material for: Enhancing Specific Energy and Power in Asymmetric Supercapacitors - A Synergetic Strategy based on the Use of Redox Additive Electrolytes
Source: Sci Rep. 2016 May 17;6:25793. doi: 10.1038/srep25793 (PMC4868987; doi:10.1038/srep25793)
Supplement: Supplementary Information [file srep25793-s1.pdf]

Supplementary Information

Enhancing Specific Energy and Power in Asymmetric Supercapacitors - A Synergetic Strategy based on the Use of Redox Additive Electrolytes

*By Arvinder Singh, Amreesh Chandra**

[*] Dr. A. Chandra

Department of Physics,

Indian Institute of Technology Kharagpur, Kharagpur-721302, West Bengal, India

E-mail: achandra@phy.iitkgp.ernet.in

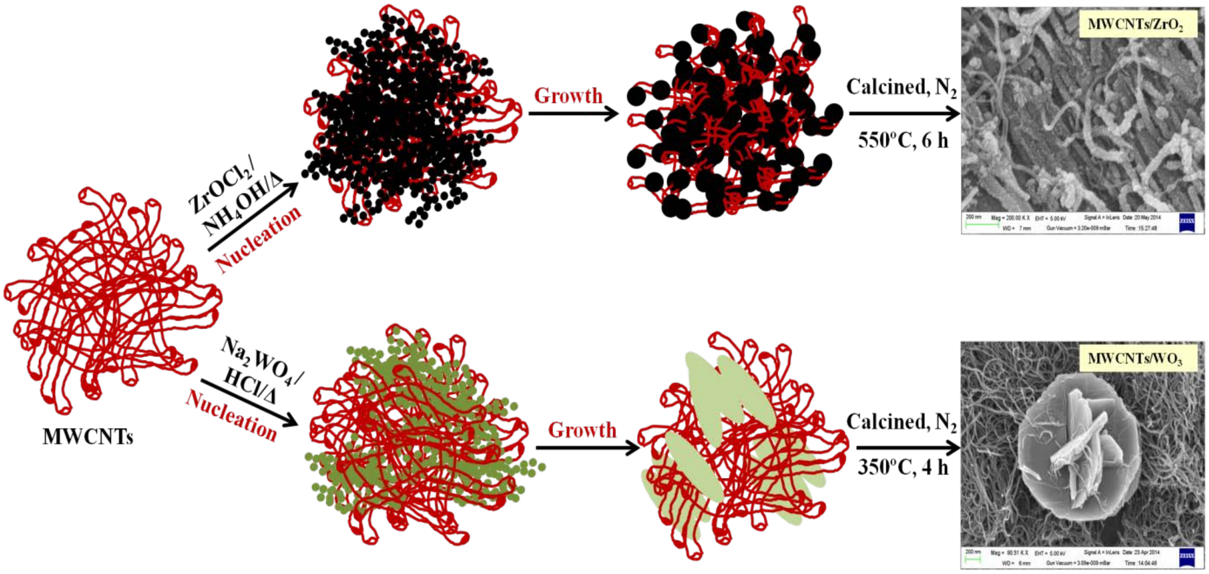

Fig. S1. Theoretically conceptualized scheme showing the steps in the synthesis of MWZ and MWW composite materials

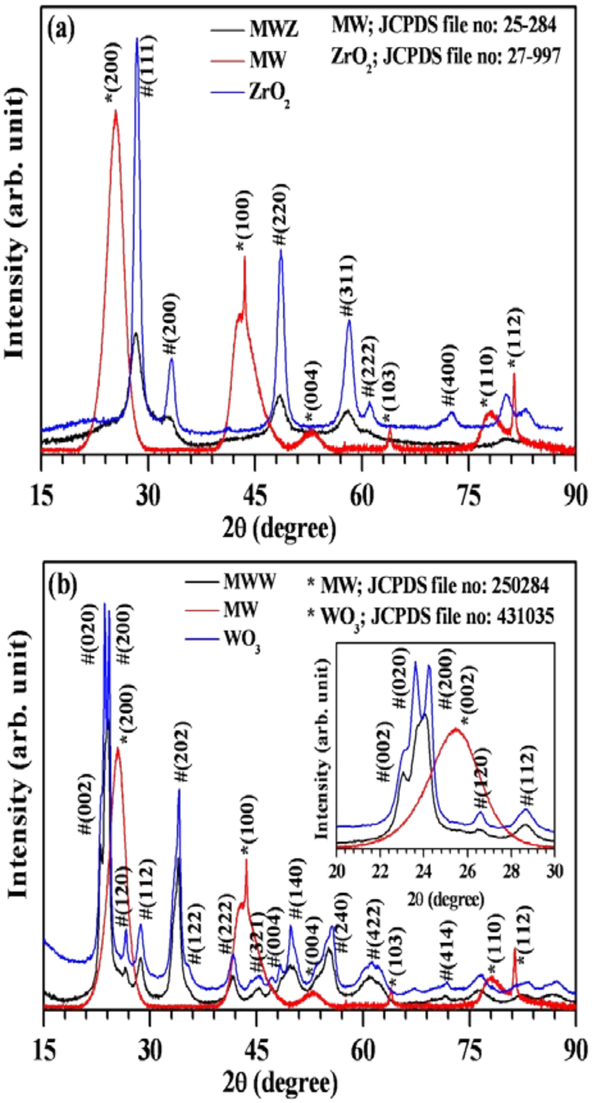

Fig. S2. XRD patterns observed for (a) MWCNTs, ZrO_2 and MWZ, (b) MWCNTs, WO_3 and MWW samples

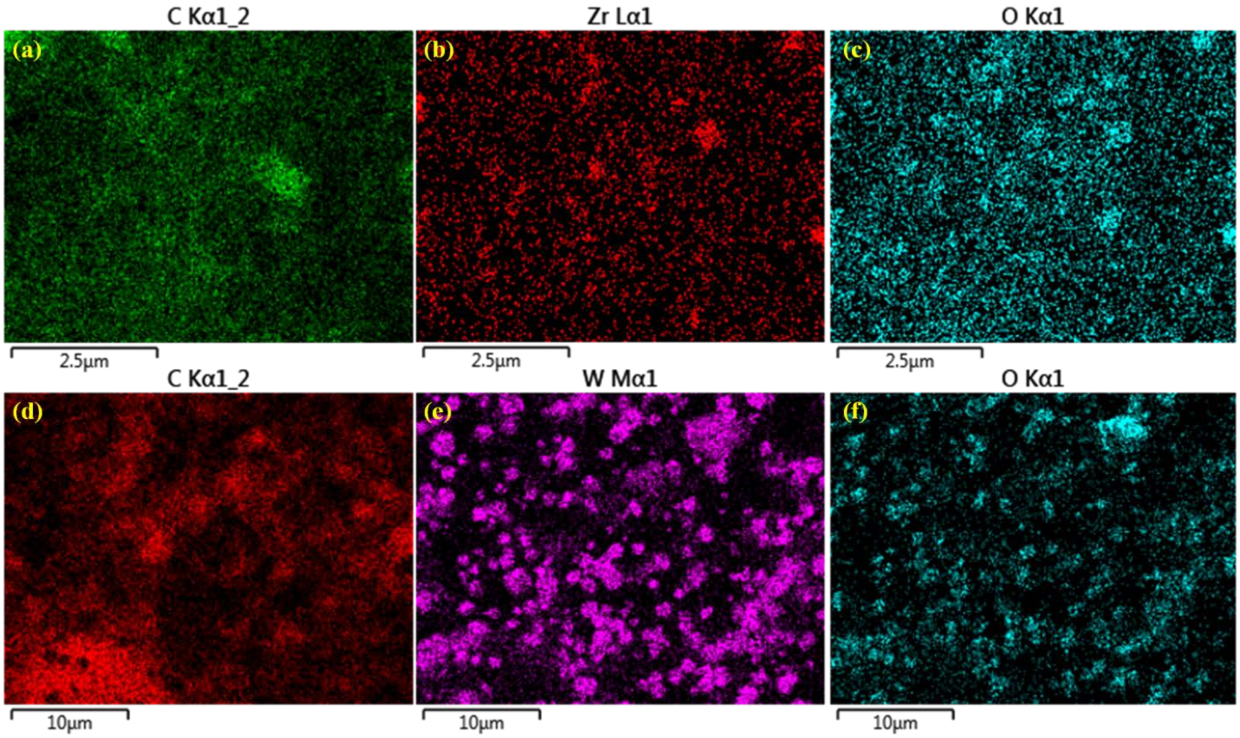

Fig. S3. Focused ion beam (FIB) elemental maps for (a) MWZ and (b) MWW composite, respectively

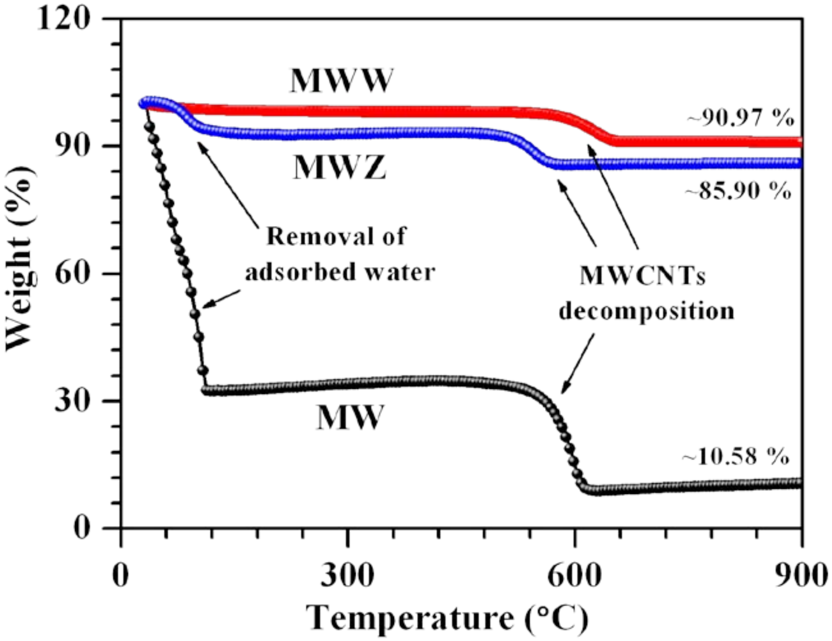

Fig. S4. Thermogravimetric analysis (TGA) curves for MWCNTs, MWZ and MWW samples

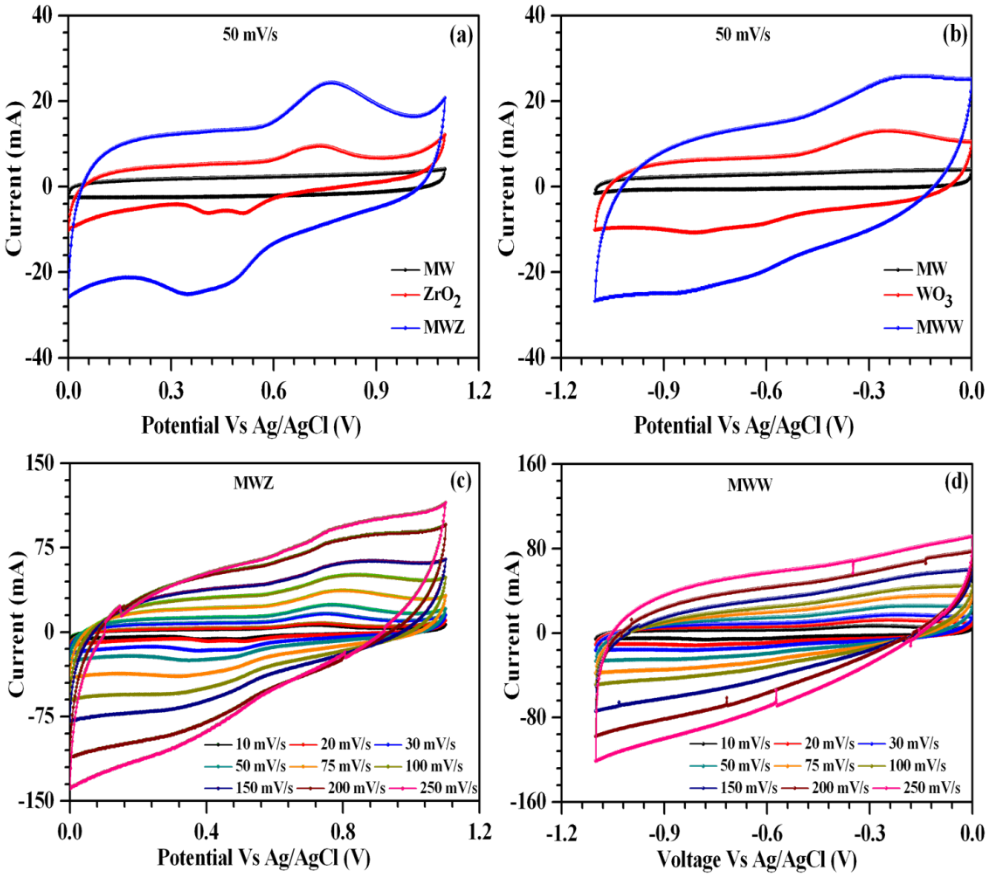

Fig. S5. Three electrode CV curves observed with Ag/AgCl (sat. KCl) as reference and Pt as counter electrode at a scan rate of 50 mV/sec for (a) MWCNTs, ZrO_2 and MWZ, (b) MWCNTs, WO_3 and MWW samples; CVs at different scan rates for (c) MWZ and (d) MWW composite material in aq. 1M Li_2SO_4 electrolyte

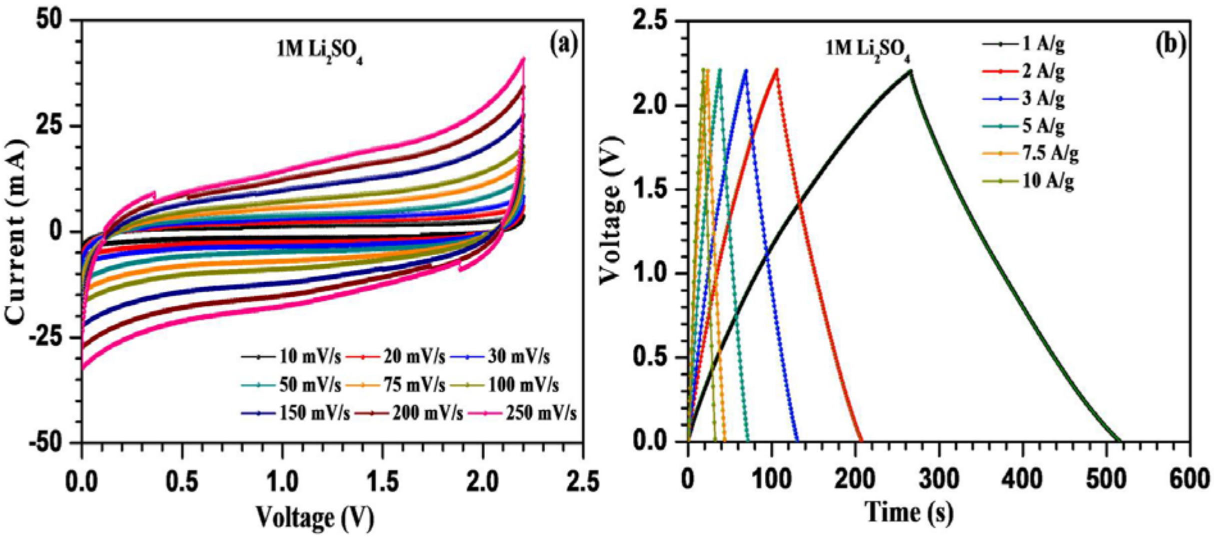

Fig. S6. (a) Two electrode CV curves observed at different scan rates (b) Galvanostatic charge-discharge curves for ASCs assembled in pure 1M Li_2SO_4 aq. electrolyte

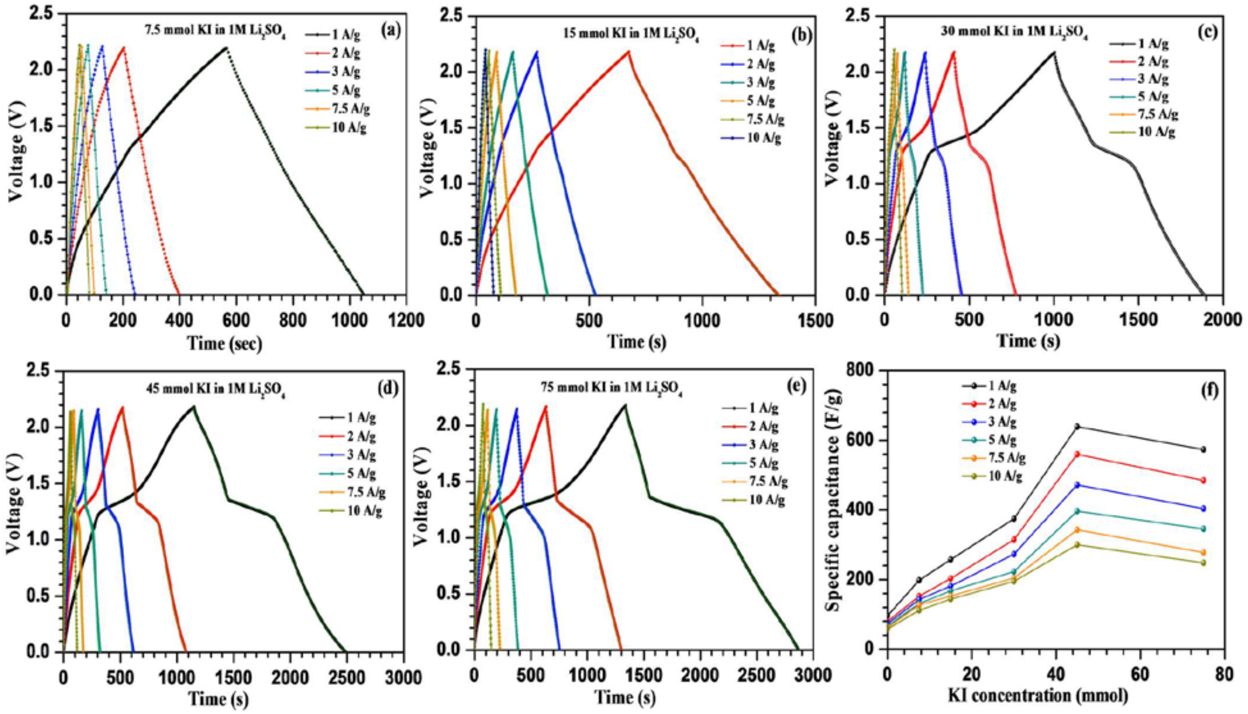

Fig. S7. Two electrode charge-discharge curves observed at different specific currents for ASCs assembled in (a) 7.5 mmol (b) 15 mmol (c) 30 mmol (d) 45 mmol and (e) 75 mmol KI added aq. 1M Li_2SO_4 electrolyte; (f) variation of specific capacitance at various specific currents for different KI concentrations

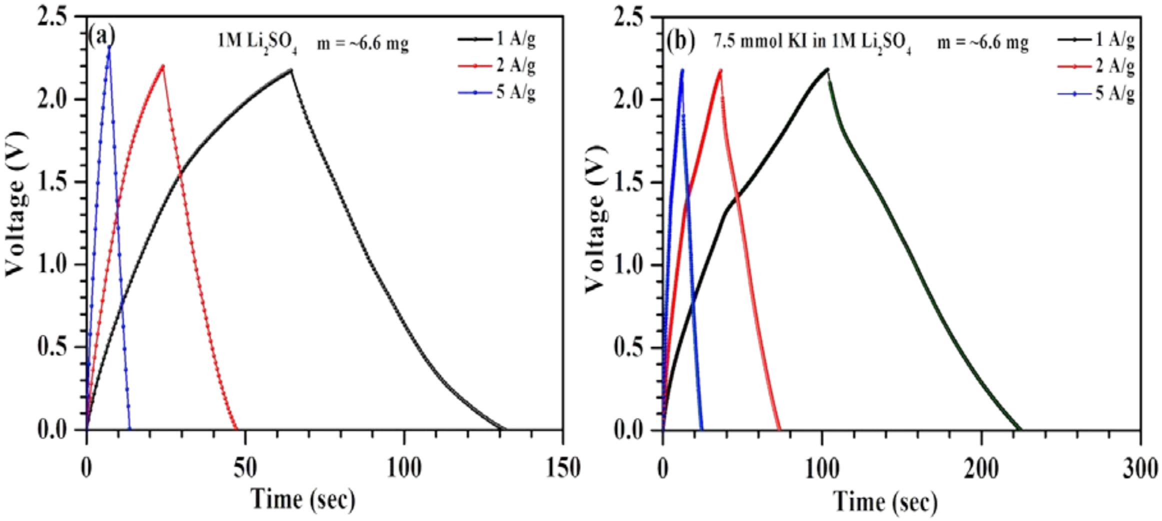

Fig. S8. Two electrode galvanostatic charge-discharge curves observed at different specific currents for ASCs with higher mass loading assembled in (a) pure and (b) 7.5 mmol KI added electrolyte systems.

Table S1: Various fitting parameters obtained from the equivalent circuit of Nyquist plots

**Electrolyte**	R_s	R_{ct}	W_o	**CPE**
**1M Li_2SO_4**	0.341 ohm	0.017 ohm	0.38	Q = 0.00078
**7.5 mmol KI addition**	0.336 ohm	0.023 ohm	0.57	Q = 0.00097
**15 mmol KI addition**	0.295 ohm	0.027 ohm	0.62	Q = 0.00126

Table S2: Performance comparison of our ASCs with previously reported aqueous ASCs

ASCs structure	Electrolyte used	Operating Voltage [V]	Specific energy [Wh/kg]	Specific power [W/kg]	Cycles and capacitance fade	Reference no
Graphene/MnO_2/fFWCNTs// activated carbon/fFWCNTs	Na_2SO_4	2.0	27	130	2000; ~5%	7
Graphene/MnO_2//Graphene	Na_2SO_4	2.0	30.4	100	1000, 21%	10
Graphene/$Ni(OH)_2$//Graphene/RuO_2	KOH	1.5	48.0	230	5000; 8%	12
3D Porous graphene/MnO_2// Graphene/Ag	Na_2SO_4	1.8	50.8	101.5	-	15
Graphene/MnO_2//Graphene/MoO_3	Na_2SO_4	2.0	42.6	276	1000; -	17
CoO/Polypyrrole//Activated carbon	NaOH	1.8	43.5	87.5	20000; 8.5%	47
$Ni(OH)_2$/Graphene//Graphene	KOH	1.6	77.8	174.7	3000; 5.7%	48
$NiMoO_4$//Activated carbon	KOH	1.7	60.9	850	10000; 4.3%	49
NiCoAl-LDH//Activated carbon	KOH	1.6	58.9	400	10000; 3%	50
CNTs/$Ni(OH)_2$//rGO	KOH	1.8	35	1800	-	51
**ZrO_2/MWCNT//WO_3/MWCNT**	**Li_2SO_4**	**2.2**	**~65**	**~950**	**1000; ~7%**	**Present**
**ZrO_2/MWCNT//WO_3/MWCNT**	**Li_2SO_4+KI**	**2.2**	**~133**	**~898**	**1000; ~10%**	**Present**
